# Serum IL-10, MMP-7, MMP-9 Levels in *Helicobacter pylori* Infection and Correlation with Degree of Gastritis

**DOI:** 10.3889/oamjms.2016.099

**Published:** 2016-09-05

**Authors:** Gontar Siregar, Sahat Halim, Ricky Sitepu

**Affiliations:** 1 *University of Sumatera Utara, Gastroentero-Hepatology, Komp. Tasbi YY No 203, Medan, Sumatera Utara 20122, Indonesia*; 2 *University of Sumatera Utara, Internal Medicine, Medan, Sumatera Utara, Indonesia*

**Keywords:** *Helicobacter pylori*, gastritis, IL-10, MMP-7, MMP-9

## Abstract

**AIM:**

*Helicobacter pylori* causes gastric mucosal inflammation and immune reaction. However, the increase of IL-10, MMP-7, and MMP-7 levels in the serum is still controversial. The objective of this study was to investigate the serum levels of IL-10, MMP-7 & MMP-9 in gastritis patients with *H. pylori* infection.

**MATERIALS AND METHODS:**

A cross-sectional study was done on seventy gastritis patients that consecutive admitted to endoscopy units. The diagnosis of gastritis was made based on histopathology and diagnosis of *H. pylori* infection was based on rapid urease test. Serum samples were obtained to determine to circulate IL-10, MMP-7, and MMP-9 level. Univariate and bivariate analysis were done by SPSS version 22.

**RESULTS:**

Forthy percentages of the patients were infected with *H. pylori*. The IL-10 level was significantly higher in *H. pylori*-infected patients compared to non-infected patients. However, there were no differences between serum levels of MMP-7 and MMP-9 in infected and non-infected *H. pylori* patients.

**CONCLUSIONS:**

The immune response to *H. pylori* promotes systemic inflammation, which was reflected by the increased levels of serum IL-10. However, there were no significant differences in MMP-7 and MMP-9 serum levels between positive and negative infected *H. pylori* patients.

## Introduction

*Helicobacter pylori* are bacteria that specifically infect the epithelial cells of the human gastric. *Helicobacter pylori* are found in the gastric of more than 50% of humans and frequently is the major cause of gastritis throughout the world [1].

The increase in cytokine production caused by *Helicobacter pylori* has been studied before, including the anti-inflammatory cytokine. This cytokine is responsible for the decrease in immune response. IL-10 is an anti-inflammatory cytokine that is capable of decreasing the inflammatory responses caused by *H. pylori*, which will cause the rise in bacterial num-bers and the effects mediated by the epithelial cells of the gaster [2]. Patients with chronic gastritis caused by *H. pylori* infection often develops peptic ulcers, as well as gastric carcinoma and lymphoma [3].

Matrix metalloproteinases (MMPs) are believed to have an essential role in the inflammation process and carcinogenesis, by causing degradation and remodelling of the extracellular matrix and basal membrane. MMP are secreted through transmembrane endoproteinases. MMP has a catalytic zinc domain, which is required for proteolytic activity. MMP are able to degrade at least one extracellular matrix component. MMP-7 and MMP-9 are members of the MMP family, which increase in *H. pylori* gastritis and in early gastric carcinoma [4].

There have been many studies on the immune response based on gastric mucosal cytokine and MMP expression in *H. pylori* infection, however, the data on levels of anti-inflammatory cytokines and MMP in the serum.

In this study, the objective is to evaluate the increase of IL-10, MMP-7 and MMP-9 in patients with *H. pylori* infection without any systemic disease and the correlation with the histopathologic degree of gastritis.

## Patients and Methods

### Patient selection

A cross-sectional study was done on seventy consecutive gastritis patients that were admitted to endoscopy units at Adam Malik General Hospital and Permata Bunda Hospital, Medan, Indonesia from May-October 2014. All patients gave informed consent and the study was approved by the local ethical committee. None of the patients had received antibiotics, bismuth compounds, H2 antagonists, proton pump inhibitors or immune modulating drugs within the last four weeks before endoscopy. Patients with evidence of malignancy, immunosuppression, metabolic disorders, or gastrointestinal haemorrhage and patients who had a history of gastric surgery were excluded [2, 5-7].

### Diagnosis of gastritis

Gastritis degree was evaluated from a biopsy of the mucosa of gastric antrum and body. The biopsy specimens were fixed in 10% formalin and embedded in paraffin. The samples were stained using Hematoxylin-Eosin and were evaluated by the pathologist of anatomic pathology referring to the visual analogue scale of the updated Sydney System. The higher degree was used if differences of degree were found between the body and antrum. The degree of chronic inflammation, neutrophil infiltration, atrophy, and intestinal metaplasia were scored 0 to 3, i.e., normal (0), mild (1), moderate (2), and severe (3) [5].

### Diagnosis of Helicobacter pylori

*H. pylori* were considered positive based on the positive results of the rapid urease test. In this study, we used Pronto Dry^®^. We used gastric antral biopsy specimen that was taken within 2 cm from the pylorus for Pronto Dry^®^. The results of the rapid urease test were read within 24 hours. The Pronto Dry^®^ was considered to be positive if the colour changed from amber to pink-red [6].

### Serum levels of IL-10, MMP-7, and MMP-9

Venous blood was drawn using a serum separator tube and allowed to clot for 30-45 minutes at room temperature before centrifugation for 15 minutes at approximately 1,000g. Serum was immediately stored frozen in aliquots at -20°C until assays for IL-10, MMP-7, and MMP-9 were performed. IL-10 was assayed by the high sensitive EBioscience technique, Bender MedSystems GmbH 1030 Vienna, Austria. Circulating MMP-7 and MMP-9 levels were examined in serum using the Quantikine Human MMP-7-ELISA and Quantikine Human MMP-9-ELISA (Quantikine, R&D System, Inc., Minneapolis). Serum levels were expressed as pg/ml. Levels above the mean were categorised as the high level, and levels below the mean were categorised as the low level.

### Statistical analysis

SPSS version 22 (SPSS Inc., Chicago) was used for analysis. The data were analysed using univariate and bivariate analysis with 95% confidence intervals. The results were expressed as the mean ± standard deviation. Bivariate analysis was carried out using the independent t-test, Mann-Whitney test, and Chi-square test with a p-value < 0.05 was considered statistically significant.

## Results

### Demographics of Respondents

There were 70 subjects, consisting of 35 males (50%) and 35 females (50%) subjects. The average age of the subjects was 49.9 ± 13.04 (SD) years. Most subjects were from the age group of 46-60 years old. 40% patients were infected with *H. pylori*. The occupation of most of the respondents was housewives (38.57%). The majority of subjects had an overweight or underweight nutritional status (58.57%) (Table 1).

**Table 1:** Characteristics of the subjects

**Variables**	***H. pylori* positive**	***H. pylori* negative**	**Frequency**
Sex Males Females	17 (24.28%) 11 (15.71%)	18 (25.71%) 24 (34.29%)	35 (50%) 35 (50%)

Age (years) <30 30-45 46-60 >60	4 (5.71%) 9 (12.86%) 9 (12.86%) 6 (8.57%)	3 (4.29%) 12 (17.14%) 18 (25.71%) 9 (12.86%)	7 (10%) 21 (30%) 27 (38.57%) 15 (21.43%)

Job Self employed Employee Farmer Housewife Other	7 (10%) 4 (5.71%) 3 (4.29%) 9 (12.86%) 5 (7.14%)	15 (21.43%) 5 (7.14%) 2 (2.86%) 18 (25.71%) 2 (2.86%)	22 (31.43%) 9 (12.85%) 5 (7.15%) 27 (38.57%) 7 (10%)

Nutritional status Normal Underweight and Overweight	9 (12.86%) 19 (27.14%)	20 (28.57%) 22 (31.43%)	29 (41.43%) 41 (58.57%)

Total	28 (40%)	42 (60%)	70 (100%)

### Serum IL-10, MMP-7 and MMP-9 levels in Helicobacter pylori infection

The circulatory IL-10 levels were significantly higher in *H. pylori*-infected patients compared to non-infected *H. pylori* patients (p < 0.005). However, there were no significant differences between serum levels of MMP-7 and MMP-9 in both groups (Table 2).

**Table 2:** Serum level of IL-10, MMP-7 & MMP-9 in HP (+) and HP (-)

Serum Level (pg/ml)	*H.pylori* positive (mean ± SD)	*H.pylori* negative (mean ± SD)	Independent t-test
IL-10	1.62 ± 1.56	1.39 ± 2.32	0.043*

MMP-7	8.69 ± 6.82	8.03 ± 6.20	0.886

MMP-9	951.86 ± 522.07	889.91 ±478.78	0.640

* p < 0.05.

### Association serum level of IL-10, MMP-7, MMP-9 and degree of gastritis

There was no correlation found between IL-10 serum levels with the degree of chronic inflammation or neutrophil infiltration. There was no correlation found between MMP-7 serum levels with the degree of chronic inflammation or neutrophil infiltration. The same results were found for MMP-9 serum levels. There was no correlation found between MMP-9 serum levels with the degree of chronic inflammation or neutrophil infiltration (Table 3).

**Table 3:** Association serum levels of IL-10, MMP-7, MMP-9 and degree of chronic inflammation and neutrophil infiltration

Cytokines		Chronic Inflammation				Neutrophil Infiltration			

		Normal + Mild N (%)	Moderate + Severe N (%)	OR (95% CI)	P	Normal + Mild N (%)	Moderate + Severe N (%)	OR (95% CI)	p
IL-10	High	15 (21.43)	19 (27.14)	1.01 (0.59-1.70)	0.978	26 (37.14)	8 (11.43)	0.872 (0.65-1.17)	0.364
	
	Low	16 (22.86)	20 (28.57)			24 (34.29)	12 (17.14)		

MMP-7	High	12 (17.14)	23 (32.86)	1.58 (0.91-2.75)	0.092	23 (32.86)	12 (17.14)	1.17 (0.87-1.58)	0.290
	
	Low	19(27.14)	16 (22.86)			27 (38.57)	8 (11.43)		

MMP-9	High	14 (20)	21 (30)	1.21 (0.72-2.06)	0.470	24 (34.29)	11 (15.71)	1.08 (0.81-1.46)	0.597
	
	Low	17 (24.29)	18 (25.71)			26 (37.14)	9 (12.86)		

There was no correlation found between IL-10 serum levels with the degree of atrophy or intestinal metaplasia. There was no correlation found between MMP-7 serum levels and the degree of atrophy or intestinal metaplasia.

The same results were found for MMP-9 serum levels. There was no correlation found between MMP-9 serum levels and the degree of atrophy or intestinal metaplasia (Table 4).

**Table 4:** Association serum levels of IL-10, MMP-7, MMP-9 and degree of atrophy and intestinal metaplasia

Cytokines		Atrophy				Intestinal Metaplasia			

		Normal + Mild N (%)	Moderate + Severe N (%)	OR (95% CI)	P	Normal + Mild N (%)	Moderate + Severe N (%)	OR (95% CI)	p
IL-10	High	27 (38.57)	7 (10)	1.13 (0.88-1.45)	0.318	25 (35.71)	9 (12.86)	1.12 (0.91-1.38)	0.276
	
	Low	32 (45.71)	4 (5.71)			30 (42.86)	6 (8.57)		

MMP-7	High	30 (42.85)	5 (7.14)	0.83 (0.65-1.07)	0.145	30 (42.86)	5 (7.14)	0.97 (0.79-1.18)	0.743
	
	Low	25 (35.71)	10 (14.28)			29 (41.43)	6 (8.57)		

MMP-9	High	29 (41.43%)	6 (8.57%)	1.04 (0.810-1.33)	0.771	27 (38.57)	8 (11.43)	1.03 (0.85-1.27)	0.743
	
	Low	30 (42.85)	5 (7.14)			28 (40)	7 (10)		

## Discussion

The average age of the subjects was 49.9 ± 13.04 (SD) years, which concludes that most of the gastritis patients are at their productive ages. The results of this study are similar to a study conducted by Garg B, et al, which reported that the average age of gastritis patients was 47 years old and the study reported by Mustapha SK, et al with an average age of 47 years old [7, 8].

In this study, a group of gastritis patients without major gastric disease (example: peptic ulcer and gastric carcinoma) were studied with the objective to examine the presence of *H. pylori* infection in gastritis patients. It is known that *H. pylori* are correlated with increased expressions of IL-10, MMP-7, and MMP-9 in the mucosa [9-12].

However, studies on the levels of IL-10, MMP-7 and MMP-9 in the serum are still limited and controversial. Owing to that, this study was conducted with the objective to find the differences between the serum levels of IL-10, MMP-7 and MMP-9 in patients with positive and negative *H. pylori*, and to analyse the correlations between the serum levels and the degree of gastritis based on histopathology appearance.

IL-10 as an anti-inflammatory cytokine has a role in the initial immune response which is mediated by B cell. Cytokine IL-10 is involved in the decrease of the inflammatory response [13]. IL-10 inhibits the synthesis of IFN-c, IL-1, IL-6, IL-8 and TNF-α, and also acts as a feedback mechanism in reducing these cytokines [13, 14]. IL-10 might contribute to the failure of the immune response to clear *H. pylori* infection, and in a previous study we found the increased secretion of IL-10 in biopsy specimens in *H. pylori* infection, with the secretion of the cytokine correlating with the degree of chronic inflammation. Yamaoka *et al* have reported increased expression of IL-10 mRNA in biopsies from infected patients [15].

IL-10 helps to maintain bacterial colonisation and exerts its effects directly on gastric epithelial cells [2, 10, 11]. Goll et al reported that samples from *H. pylori*-positive patients showed increased production of IL-10 as much as 6.7 times the production of patients with negative *H. pylori*.

In this study, the serum levels of IL-10 increased significantly in positive *H. pylori* compared to negative *H. pylori* patients. This result was similar to that reported by Dlugovittzky et al, where the IL-10 serum levels of the positive *H. pylori* subjects were higher compared to negative *H. pylori* subjects (p<0.01) [16]. However, other studies reported different conclusions, In the studies performed by Russo et al and Bayraktaroglu et al, they reported that there were no significant correlations between the positive *H. pylori* subjects and negative subjects, which might be caused by inhibition of further inflammation [17, 18].

*H. pylori* is able to trigger the expression of MMP, which is a proteolytic enzyme that has a role in maintaining and remodelling the interaction between epithelial cells and the basal membrane [19, 20]. A study, conducted by Bebb JR *et al*, found that gastric biopsy specimens from a positive *H. pylori* subject will show in a higher level of MMP-7 protein in the antrum and corpus [19]. A similar result is found from the study conducted by Wroblewski *et al*, which concluded that the MMP-7 expression in the antrum and corpus increased with the presence of *H. pylori* [21].

In this study, there were no significant differences in MMP-7 serum levels between positive and negative *H. pylori* subjects. The same results were found in the study by Rautelin et al, which concluded that there were no significant differences in MMP-7 serum levels between positive and negative *H. pylori* gastritis subjects [22].

Studies by Rautelin HI *et al* and Li SL *et al* found that MMP-9 level increased significantly in the gastric mucosa of gastritis patients with positive *H. pylori* compared to the negative [22, 23]. In a study conducted by Oliviera, it was found that *H. pylori* through a T4SS (Type IV Secretion System) pathway increases the activity of MMP-9 as a response to the invasion of *H. pylori* on the gastric epithelium [24].

In this study, there was no difference in the serum levels of MMP-9 between the positive and negative *H. pylori* patients. Similar results were also found in the study reported by Rautelin et al and Ettehad et al, where it was concluded that there were no significant differences in the MMP-9 levels of the gastritis patients between the positive and negative *H. pylori* subjects [25, 26].

In this study, it was found that there were no significant correlations found between serum levels of IL-10, MMP-7, MMP-9 and the degree of chronic inflammation, neutrophil infiltration, atrophy, or intestinal metaplasia (p>0.05).

In conclusion, the IL-10 serum levels increased significantly in positive *H. pylori* subjects. There was no significant difference in the average serum levels of MMP-7 and MMP-9 between positive and negative infected *H. pylori* patients. There were no correlations found between serum levels of IL-10, MMP-7, MMP-9 with the degree of gastritis based on histopathology.
